# Current Status and Future Directions of Capsule Endoscopy

**DOI:** 10.1155/2016/4159745

**Published:** 2016-01-28

**Authors:** Hoon Jai Chun, Satoshi Tanabe, Myung-Gyu Choi, Jean-Christophe Saurin

**Affiliations:** ^1^Division of Gastroenterology and Hepatology, Department of Internal Medicine, Institute of Gastrointestinal Medical Instrument Research, Korea University College of Medicine, Inchon-ro 73, Seongbuk-gu, Seoul 02841, Republic of Korea; ^2^Division of Therapeutic Endoscopy, Department of Advanced Medicine, Research and Development Center for New Medical Frontiers, Kitasato University School of Medicine, 1-15-1 Kitazato, Minami-ku, Sagamihara, Kanagawa 252-0374, Japan; ^3^Division of Gastroenterology, Department of Internal Medicine, Seoul St. Mary's Hospital, College of Medicine, The Catholic University of Korea, 222 Banpo-daero, Seocho-gu, Seoul 06591, Republic of Korea; ^4^Edouard Herriot Hospital, Gastroenterology Unit, Pavillon L, 5 Place d'Arsonval, 69437 Lyon Cedex 03, France

Small bowel capsule endoscopy (CE) is a method of endoluminal examination of the small bowel using a wireless disposable capsule-shaped tool which is swallowed and then propelled by gut motility through the gastrointestinal tract. Since its first use more than 10 years ago, CE has been a first-line diagnostic method for the evaluation of the small bowel. The most common indications for CE include obscure gastrointestinal bleeding (OGIB), suspected Crohn's disease, small bowel tumor, and polyposis syndrome. Up until now, the application of CE has been widened based on the results of many clinical studies.

In the current special issue, issues other than OGIB, such as Crohn's disease, ileitis, and portal-hypertensive enteropathy (PHE), have been focused on. However, CE has some limitations, such as inadequate bowel preparation and incompletion. To increase diagnosis yield, adequate bowel preparation and complete examination of the entire small bowel are important. And another important limitation of CE is the risk of retention. As one of the efforts to solve this problem, the real clinical experience with the PillCam patency capsule prior to CE was introduced.

D.-H. Yang et al. have extensively reviewed the diagnostic accuracy and the therapeutic impact of CE in Crohn's disease. However, clinical experience with CE for the diagnosis and management of Crohn's disease is still limited despite the advantages of CE over other modalities. The risk of capsule retention, lack of evidence on the clinical benefit, and lack of established indications for CE in Crohn's disease may limit the widespread use of CE in clinical practice for suspected or established Crohn's disease.

Ileitis is defined as inflammation of the ileum, and various etiologies are associated with ileitis. However, the differential diagnosis of terminal ileitis can be difficult in many cases. H. S. Lee and Y. J. Lim have described each of the various conditions associated with ileitis and the diagnostic value of CE for ileitis, which may help identify and evaluate these conditions in clinical practice.

S. R. Jeon and J.-O. Kim have reviewed that CE has enabled the identification of PHE as a potentially significant complication of portal hypertension. In patients with liver cirrhosis and OGIB, PHE might be a possible cause for GI blood loss. CE can be a useful method for identifying treatable lesions in patients with PHE and can help optimize treatment on a case-by-case basis.

Bowel preparation before CE is as essential as bowel preparation before colonoscopy. To date, there have been many comparative studies, consensus, and guidelines regarding different kinds of bowel cleansing agents in bowel preparation for small bowel CE. H. J. Song et al. have reviewed previous studies regarding bowel preparation for small bowel CE and suggested optimal bowel preparation of VCE.

C. Römmele et al. have performed a retrospective analysis of 38 patients with risk factors for capsule retention and have indicated that CE could safely be performed even if the patency capsule excretion time is longer than 30 hours and the excreted patency capsule was not screened for damage.

